# Prognostic biomarker NEIL3 and its association with immune infiltration in renal clear cell carcinoma

**DOI:** 10.3389/fonc.2023.1073941

**Published:** 2023-02-02

**Authors:** Xiaomei Sun, Pengfei Liu

**Affiliations:** ^1^ Graduate School, Tianjin University of Traditional Chinese Medicine, Tianjin, China; ^2^ Department of Medical Oncology, Tianjin Academy of Traditional Chinese Medicine Affiliated Hospital, Tianjin, China

**Keywords:** NEIL3, biomarker, kidney renal clear cell carcinoma, immune infiltration, prognosis

## Abstract

**Background:**

Kidney renal clear cell carcinoma (KIRC) is a malignant tumor with a high degree of immune infiltration. Identifying immune biomarkers is essential for the treatment of KIRC. Studies have identified the potential of NEIL3 to modulate the immune microenvironment and promote tumor progression. However, the role of NEIL3 in KIRC remains uncertain. This study was to investigate the effect of NEIL3 on the prognosis and immune infiltration of patients with KIRC.

**Methods:**

TCGA and GEO databases were used to study the expression of NEIL3 in KIRC. Cox regression analysis was used to examine the relationship between the expression of NEIL3 and clinicopathological variables and survival. Furthermore, Gene Set Cancer Analysis (GSCA) was applied to study the impact of NEIL3 methylation on outcomes of KIRC. Through gene ontology (GO) and Gene set enrichment (GSEA) analysis, the biological processes and signal pathways related to NEIL3 expression were identified. In addition, immune infiltration analysis was conducted *via* CIBERSORT analysis, ssGSEA analysis and TISIDB database.

**Results:**

NEIL3 was overexpressed in KIRC, and it was significantly related with histologic grade, pathologic stage, T stage, M stage, and vital status of KIRC patients (*P* < 0.001). The expression of NEIL3 was associated with worse outcomes. Univariate and multivariate Cox analysis showed that NEIL3 may be an indicator of adverse outcomes in KIRC. GSEA analysis revealed that NEIL3 may be involved in signal pathways including cell cycle, DNA replication, mismatch repair, P53 signal pathway, and antigen processing and presentation. In addition, immune infiltration analysis showed a positive correlation between NEIL3 expression and multiple immune cells (activated CD8 T cells, activated dendritic cells, myeloid-derived suppressor cells, follicular helper T cells, and regulatory T cells) and immunoinhibitors (PD1, CTLA4, LAG3, TIGHT, IL10, and CD96).

**Conclusion:**

NEIL3 is a potential independent biomarker of KIRC, which is relevant to immune infiltration.

## Introduction

Renal cell carcinoma (RCC) is a common malignancy and accounts for more than 90% of kidney tumors, of which kidney renal clear cell carcinoma (KIRC) is the most prevalent, accounting for about 60%-85% of kidney cancers (1). According to epidemiological statistics, RCC accounts for 2% of cancer-related diagnoses and deaths worldwide, and the incidence of RCC has continued to increase in most countries and regions over the past few decades (2, 3). With the rapid development of targeted therapy and immunotherapy in the past decade, the 5-year survival rate of RCC patients, especially those with early local lesions, has improved significantly, but the overall prognosis remains poor (4, 5). In addition, the widespread use of targeted drugs and immunological agents has inevitably led to drug resistance problems (6). Therefore, it is essential to develop some effective biomarkers to assess the prognosis of patients and their response to treatment.

Endonuclease VIII-like 3 (NEIL3) is a monofunctional glycosylase involved in DNA interstrand crosslink damage repair and has an important role in maintaining telomere integrity (7, 8). Previous studies have shown that NEIL3 can repair oxidative telomere damage during the S phase, and deletion of NEIL3 can lead to telomere fusion, translocation, and loss, which indicates that NEIL3 serves a pivotal role in DNA repair (9). NEIL3 deficiency is also associated with increased lymphocyte apoptosis and autoimmune susceptibility (10). A study has found that NEIL3 can prevent senescence in hepatocellular carcinoma by repairing oxidative damage to telomeres during mitosis, and it is associated with poor prognosis (11). The overexpression of NEIL3 is also related to metastasis and poor prognosis in melanoma patients (12). In addition, there is evidence that NEIL3 is correlated with astrocytoma progression, drug resistance, and survival prognosis (13). However, the impact of NEIL3 on the prognosis and immunotherapy in patients with KIRC is poorly understood.

In this study, we investigated the relationship between NEIL3 expression and clinical features and outcomes of KIRC patients. Then, we explored the potential biofunctions of NEIL3 through GO analysis, and identified the carcinogenic and immune-related pathways using GSEA analysis. In addition, we investigated the impact of NEIL3 methylation on survival in KIRC patients. Finally, we also evaluated the connection between NEIL3 and immune infiltration levels and immunomodulators.

## Materials and methods

### Data source and preprocessing

Firstly, the expression level of NEIL3 in pan-cancer, including KIRC,was investigated by using the Tumor Immune Estimation Resource (TIMER.0) database (http://timer.cistrome.org/) (14). Level 3 RNA-sequencing data (HTSeq-Counts and HTSeq-FPKM) and clinical information of KIRC were downloaded from the UCSC Xena browser (https://xenabrowser.net/) (15). We converted HTSeq-FPKM data into transcripts per million (TPM) format for further analyses, and used HTSeq-Counts for differential analysis by the DESeq2 package. Cases with complete clinical characteristics and survival information were included in the study.

Microarray data of KIRC expression profiling (GSE40435, GSE53757 (16), GSE68417 (17) and GSE73731) were obtained from Gene Expression Omnibus (GEO) database (https://www.ncbi.nlm.nih.gov/geo/) (18). All gene expression microarray data were normalized using the limma R package. GSE40435, GSE53757 and GSE68417 were used to validate the differential expression of NEIL3 in clear cell carcinoma and normal tissues. GSE73731 with 265 KIRC samples was used to verify the relationship between NEIL3 expression and immune infiltration.

### Survival analysis and prognostic model construction

Clinical parameters of KIRC patients, including age, sex, histological grade, pathological stage, TNM stage, and vital status, were selected from previously downloaded clinical information for further analysis. Subsequently, based on median NEIL3 expression, we classified the patients into two groups. The survival curves were plotted to compare overall survival (OS), progression-free interval (PFI), and disease-specific survival (DSS) between two groups by the R package survminer (19). The receiver operating characteristic (ROC) curve was drawn by the R package pROC to verify the diagnostic value of NEIL3. We identified risk factors by univariate and multivariate Cox analysis. Furthermore, we construct a nomogram model by using the R package rms. The calibration curves were plotted to compare the consistency of the model prediction probability with the observed probability. The time-dependent ROC was performed by using the time ROC package to evaluate the prediction accuracy of the model. Moreover, we performed a decision curve analysis (DCA) to verify the clinical value of the predictive model.

### Functional enrichment analysis

Gene Ontology (GO) analysis was performed to predict NEIL3-related biological functions using the R package clusterProfiler (20). GSEA v4.2.3 software (21) was used for gene set enrichment analysis (GSEA) to identify NEIL3-related signal pathways. For each analysis, we set up 1000 permutations. The normalized enrichment score (NES) and false discovery rate (FDR) were applied to select significant pathway enrichment.

### Co-expression network analysis

To further investigate the molecular mechanisms associated with NEIL3, we usedthe GEPIA browser (http://gepia.cancer-pku.cn/) (22) to identify co-expressed genes significantly associated with NEIL3. Subsequently, Spearman’s correlation analysis was carried out on the co-expressed genes. Meanwhile, the correlation between co-expressed genes and NEIL3 was also validated in the TIMER2.0 database. In addition, a protein-protein interaction (PPI) network was established through the STRING database (https://cn.string-db.org/) (23).

### Methylation analysis

Gene Set Cancer Analysis (GSCA) (http://bioinfo.life.hust.edu.cn/GSCA/#/) is a comprehensive platform, which can provide methylation analysis (24). Thus, we used the data from GSCA online software to analyzed the relationship between NEIL3 methylation and survival in KIRC patients.

### Immune infiltration analysis

CIBERSORT was applied to calculate the proportion of 22 immune cells in KIRC patients (25). We performed single-sample Gene Set Enrichment Analysis (ssGSEA) to evaluate the relationship between NEIL3 expression and 28 immune cells by the R package GSVA (26). In addition, in order to predict the response of KIRC patients to immunotherapy, we also used TISIDB database (http://cis.hku.hk/TISIDB/) (27) to analyze the correlation between NEIL3 expression and immunomodulators.

### Statistical analysis

All statistical analyses were performed by R (4.1.3). Chi-square or Fisher’s exact test was used to compare the clinical characteristics between high expression group and low expression group. Spearman’s coefficient was used for correlation analysis. The association between clinicopathological features and NEIL3 expression was analyzed using the Wilcoxon signed-rank sum test and logistic regression. In this study, all hypothetical tests were bilateral, and *P* < 0.05 was considered to be statistically significant.

## Results

### The overexpression of NEIL3 in KIRC

**Figure 1:**
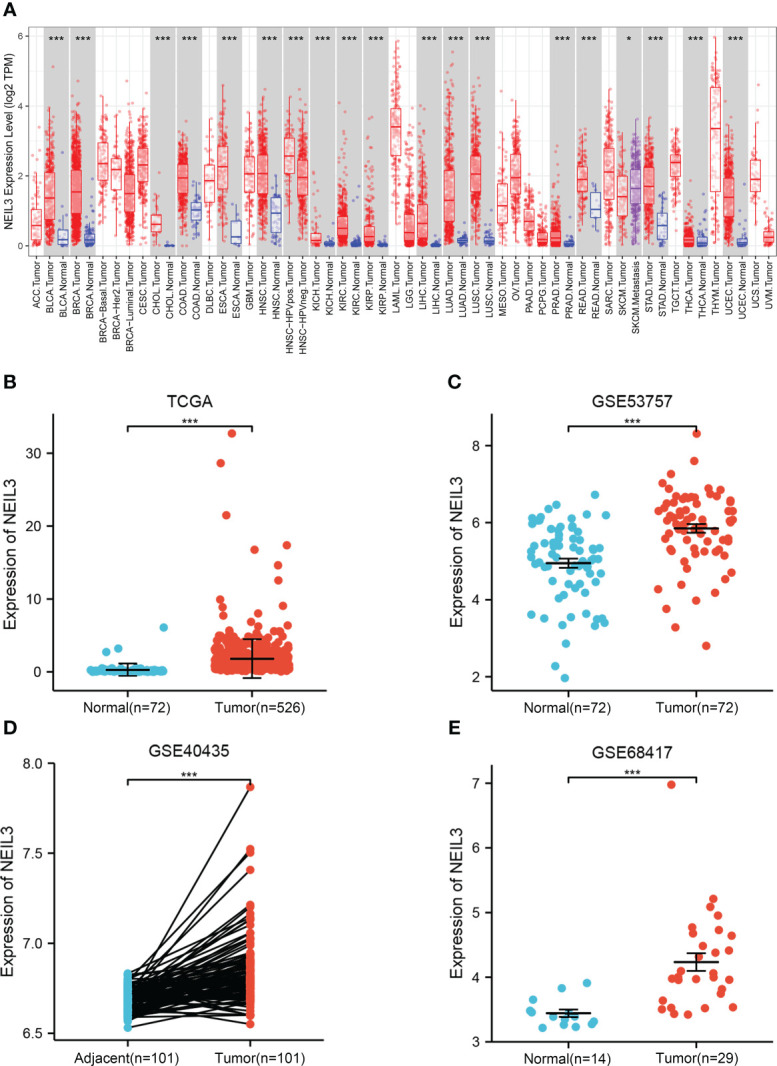
The expression level of NEIL3 is upregulated in KIRC. **(A)** Differential expression of NEIL3 between different tumors and adjacent normal tissues. NEIL3 is overexpressed in kidney renal clear cell carcinomas (KIRC), urothelial bladder carcinoma (BLCA), breast cancer (BRCA), colon adenocarcinoma (COAD), esophageal carcinoma (ESCA), liver hepatocellular carcinoma (LIHC), lung adenocarcinoma (LUAD). **(B)** NEIL3 expression level between KIRC tissues and normal tissue in TCGA. **(C–E)** Comparison of NEIL3 expression level between tumor tissues and normal or adjacent tissues in the GSE53757 **(C)**, GSE40435 **(D)**, and GSE68417 **(E)** datasets. *, *P* < 0.05; ***, *P* < 0.001.

Firstly, we assessed NEIL3 expression levels in different human tumors using the TIMER database. We found that NEIL3 was over-expressed in multiple tumors, including KIRC (**Figure 1A**). In addition, we further evaluated the expression levels of NEIL3 in KIRC using TCGA and GEO data, and found that the NEIL3 expression level in KIRC tissues was remarkably higher compared with that in normal kidney tissues (all *P* < 0.001) (**Figures 1B–E**).

### Association between NEIL3 expression and clinical features

To investigate the connection between clinical parameters and NEIL3 expression, our study incorporated clinical information of 539 KIRC samples from the TCGA database. Based on the median NEIL3 expression level, we divided these samples into two groups (low expression and high expression group). The results suggested that NEIL3 expression was closely related to histologic grade, pathologic stage, T stage, M stage, and vital status (all *P* < 0.001) (**Table 1**). However, age (*P* = 0.698), gender (*P* = 0.953), and N stage (*P* = 0.062) had no correlation with NEIL3 expression (**Table 1**).

**Table 1:** Correlation between NEIL3 expression and clinicopathological variables in patients with KIRC.

Characteristic	Low expression of NEIL3	High expression of NEIL3	*P*
n	269	270	
Age, n (%)			0.698
<=60	137 (25.4%)	132 (24.5%)	
>60	132 (24.5%)	138 (25.6%)	
Gender, n (%)			0.953
Female	92 (17.1%)	94 (17.4%)	
Male	177 (32.8%)	176 (32.7%)	
Histologic grade, n (%)			< 0.001
G1	11 (2.1%)	3 (0.6%)	
G2	134 (25.2%)	101 (19%)	
G3	95 (17.9%)	112 (21.1%)	
G4	23 (4.3%)	52 (9.8%)	
Pathologic stage, n (%)			< 0.001
Stage I	157 (29.3%)	115 (21.5%)	
Stage II	33 (6.2%)	26 (4.9%)	
Stage III	54 (10.1%)	69 (12.9%)	
Stage IV	25 (4.7%)	57 (10.6%)	
T stage, n (%)			< 0.001
T1	160 (29.7%)	118 (21.9%)	
T2	39 (7.2%)	32 (5.9%)	
T3	69 (12.8%)	110 (20.4%)	
T4	1 (0.2%)	10 (1.9%)	
N stage, n (%)			0.062
N0	111 (43.2%)	130 (50.6%)	
N1	3 (1.2%)	13 (5.1%)	
M stage, n (%)			< 0.001
M0	225 (44.5%)	203 (40.1%)	
M1	23 (4.5%)	55 (10.9%)	
OS event, n (%)			< 0.001
Alive	205 (38%)	161 (29.9%)	
Dead	64 (11.9%)	109 (20.2%)	
Age, median (IQR)	60 (52, 69)	61 (52, 71)	0.715

P < 0.05, statistically significant.

### Prognostic analysis of NEIL3 in KIRC

**Figure 2:**
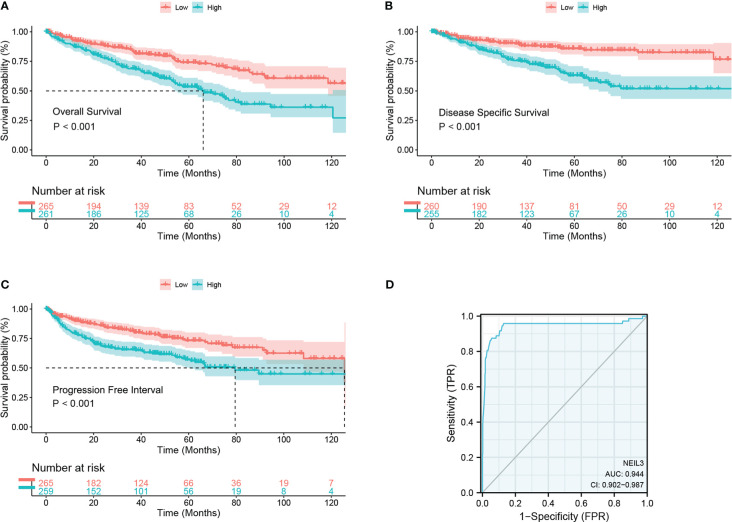
High NEIL3 expression is correlated with worse survival. **(A–C)** Survival curves of overall survival **(A)**, disease-specific survival **(B)**, and progression-free stage **(C)** between NEIL3-high and -low groups. **(D)** Diagnostic ROC of NEIL3.

**Figure 3:**
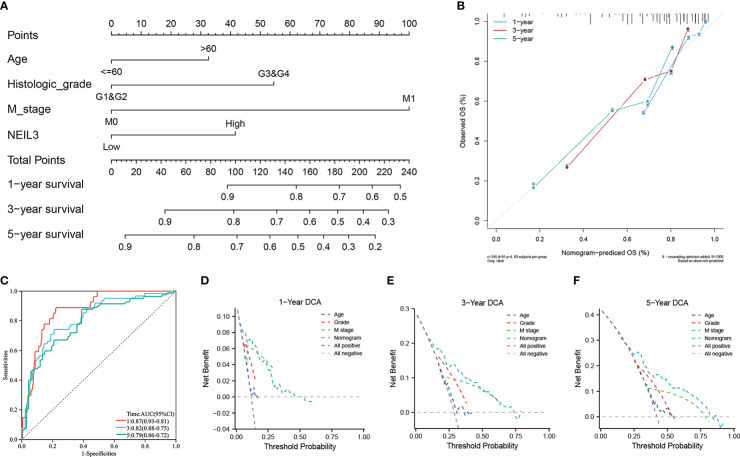
The role of NEIL3 in predicting the probability of survival in patients with KIRC. **(A)** A nomogram for predicting the probability of 1-, 3-, and 5- year OS. **(B)** Calibration curves. **(C)** Time dependent ROC curves. **(D–F)** DCA curves of 1-, 3-, and 5-year OS predicted by the nomogram and clinical variables.

Survival analysis showed that up-regulation of NEIL3 was relevant to worse OS, DSS, and PFI in KIRC patients (**Figures 2A–C**). Moreover, we performed ROC analysis to assess the diagnostic value of NEIL3 to identify KIRC tissues, and found that the area under curve (AUC) value was 0.944 (**Figure 2D**). The result indicated that NEIL3 had a strong diagnostic performance. In addition, to further explore the risk factors for patients with KIRC, we conducted univariate and multivariate analyses. Univariate Cox regression analysis revealed that age, histologic grade, pathologic stage, TNM stage, and NEIL3 expression were associated with OS (**Table 2**). Multivariate Cox survival analysis showed that age (*P* = 0.046), histologic grade, distant metastasis (*P* < 0.001) and NEIL3 expression (*P* = 0.009) were independent predictors of poor prognosis in KIRC patients (**Table 2**). Based on the multivariate cox regression analysis, a nomogram model was created to predict the probability of survival using NEIL3 expression levels, age, histologic grade, and M stage (**Figure 3A**). The calibration curves showed good agreement between the predicted OS and the observed OS at 1, 3, and 5 years (**Figure 3B**). The time ROC curve showed that the nomogram model could effectively predict the OS of KIRC patients at 1, 3, and 5 years (**Figure 3C**). Additionally, we obtained a consistency index (C-index) of 0.753. These results indicated that this model had a relatively reliable predictive performance.

**Table 2:** Univariate analysis and multivariate analysis of the prognostic factors in KIRC patients.

Characteristics	HR (95%CI) Univariate analysis	*P* value Univariate analysis	HR (95%CI) Multivariate analysis	*P* value Multivariate analysis
Age (>60 vs. ≤60)	1.54 (1.01-2.36)	0.046	1.55 (1.01-2.39)	0.046
Gender (Male vs. Female)	1.05 (0.69-1.60)	0.833		
Grade (G3&G4 vs. G1&G2)	2.65 (1.66-4.23)	<0.001	1.72 (1.05-2.83)	0.032
Stage (III&IV vs. I&II)	3.48 (2.23-5.42)	<0.001	1.08 (0.42-2.77)	0.876
T stage (T3&T4 vs. T1&T2)	3.05 (2.00-4.66)	<0.001	1.87 (0.82-4.28)	0.138
N stage (N1 vs. N0)	3.09 (1.60-5.98)	0.001	1.50 (0.74-3.02)	0.259
M stage (M1 vs. M0)	4.11 (2.66-6.37)	<0.001	2.98 (1.75-5.09)	<0.001
NEIL3 (High vs. Low)	1.72 (1.13-2.63)	0.012	1.79 (1.16-2.77)	0.009

CI, confidence interval. P < 0.05, statistically significant.

In addition, we performed DCA analysis to verify the clinical practicability of the predictive model. The DCA curves revealed that the combined model had a higher net benefit in predicting OS at 1, 3, and 5 years compared to other models that included a single clinical variable (**Figures 3D–F**). The results indicated that the combination of NEIL3 with clinical features was beneficial to predict the prognosis of KIRC patients.

### Functional enrichment analysis of NEIL3

**Figure 4:**
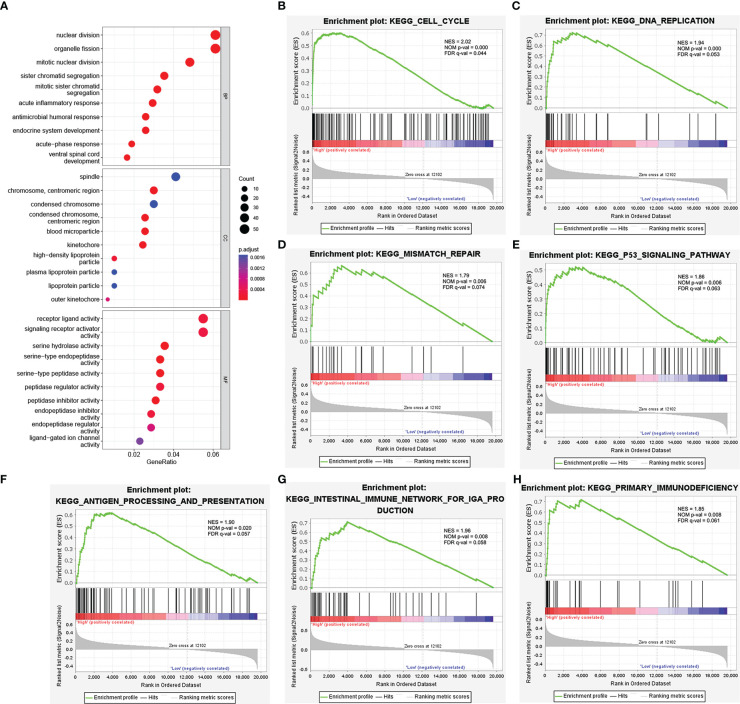
Functional enrichment analysis of NEIL3. **(A)** Enrichment plot from the GO analysis. Some biological processes (BP), cellular components (CC), and molecular functions (MF) were associated with NEIL3-related genes. **(B–H)** Enrichment plots from gene set enrichment analysis (GSEA). The GSEA results showed that the terms “Cell cycle”, “DNA replication”, “Mismatch repair”, “p53 signal pathway”, “Antigen processing and presentation”, “Intestinal immune network for IgA production”, and “Primary immunodeficiency” were differentially enriched in NEIL3 high expression phenotype. NES, normalized enrichment score; NOM p-value, nominal *P* value; FDR, false discovery rate.

Next, we conducted a GO analysis to characterize the biological functions of NEIL3 in KIRC. The GO analysis showed that NEIL3 was engaged in multiple biological processes, including nuclear division, mitotic nuclear division, acute inflammatory response, kinetochore, chromosome condensation, serine hydrolase activity, and receptor-ligand activity signal pathway (**Figure 4A**). In addition, to further investigate NEIL3-related signal pathways in renal clear cell carcinogenesis, we performed GSEA analysis and found 16 signal pathways demonstrated significant differences (NES > 1.72, *P*-value < 0.05, FDR < 0.1) in the high expression group (**Table 3**). Cell cycle, DNA replication, mismatch repair, and p53 signal pathway are the important signal pathways involved in tumorigenesis in the high NEIL3 group, while the signal pathways involved in the immune response included antigen processing and presentation, intestinal immune network for IgA production, primary immunodeficiency (**Figures 4B–H**).

**Table 3:** Gene sets enriched in the high NEIL3 expression group.

GS follow link to MSigDB	SIZE	NES	NOM p-val	FDR q-val
KEGG_BASE_EXCISION_REPAIR	33	2.02	0.002	0.083
KEGG_CELL_CYCLE	124	2.02	0.000	0.044
KEGG_INTESTINAL_IMMUNE_NETWORK _FOR_IGA_PRODUCTION	46	1.96	0.008	0.058
KEGG_DNA_REPLICATION	36	1.94	0.000	0.053
KEGG_SYSTEMIC_LUPUS_ERYTHEMATOSUS	54	1.90	0.018	0.065
KtEGG_ANTIGEN_PROCESSING_AND_PRESENTATION	80	1.90	0.020	0.057
KEGG_HOMOLOGOUS_RECOMBINATION	28	1.86	0.006	0.072
KEGG_P53_SIGNALING_PATHWAY	67	1.86	0.006	0.063
KEGG_PRIMARY_IMMUNODEFICIENCY	35	1.85	0.008	0.061
KEGG_AUTOIMMUNE_THYROID_DISEASE	50	1.83	0.015	0.063
KEGG_TYPE_I_DIABETES_MELLITUS	41	1.81	0.028	0.068
KEGG_PYRIMIDINE_METABOLISM	97	1.79	0.004	0.074
KEGG_MISMATCH_REPAIR	23	1.79	0.006	0.074
KEGG_VIRAL_MYOCARDITIS	68	1.77	0.026	0.079
KEGG_ALLOGRAFT_REJECTION	35	1.75	0.030	0.088
KEGG_LEISHMANIA_INFECTION	69	1.75	0.040	0.084

NES, normalized enrichment score; NOM, nominal; FDR, false discovery rate. Gene sets with NOM p-value < 0.05 and FDR q-value < 0.1 are considered as significant.

### Analysis of genes coexpressed with NEIL3

**Figure 5:**
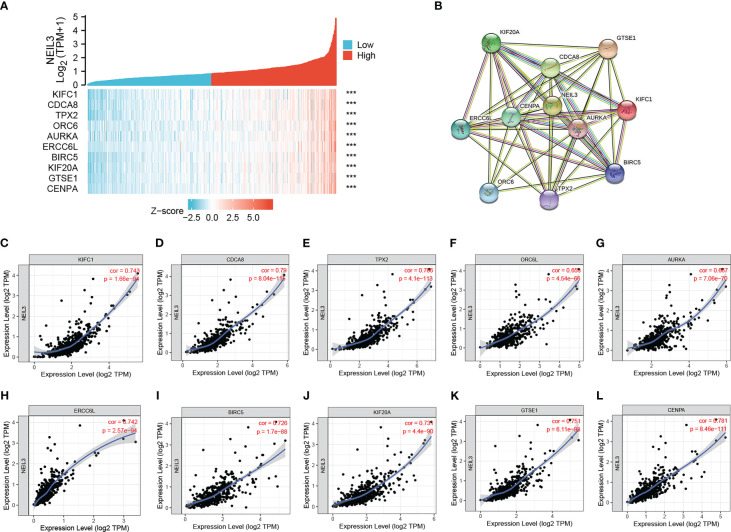
Correlation analysis of genes coexpressed with NEIL3. **(A)** The top ten genes positively correlated with NEIL3 were shown in the heatmap. **(B)** The PPI network of the top ten genes was established using STRING. **(C–L)** The correlation between NEIL3 and related genes was analyzed by the TIMER.0 database. Results show that NEIL3 is remarkably relevant to KIFC1 (cor = 0.743, *P* = 1.66e-94), CDCA8 (cor = 0.79, *P* = 8.04e-115), TPX2 (cor = 0.786, *P* = 4.1e-113), ERCC6L (cor = 0.742, *P* = 2.57e-94), BIRC5 (cor = 0.726, *P* = 1.7e-88), KIF20A (cor = 0.731, *P* = 4.4e-90), GTSE1 (cor = 0.751, *P* = 6.11e-98), CENPA (cor = 0.781, *P* = 8.46e-111), ORC6 (cor = 0.653, *P* = 4.54e-66) and AURKA (cor = 0.667, *P* = 7.06e-70). ORC6L, Aliases for ORC6 Gene. ***, *P* < 0.001.

In order to further explore the potential role of NEIL3 in renal clear cell carcinogenesis, we identified genes that were positively correlated with NEIL3 through the GEPIA database. The top ten related genes (r ≥ 0.73) are shown in the co-expression heatmap (**Figure 5A**). The interaction network for these genes was established by using the STRING database (**Figure 5B**). In addition, the correlation between NEIL3 and these genes was validated in the TIMER2.0 database. The analysis suggested that NEIL3 was significantly associated with KIFC1 (cor = 0.743, *P* = 1.66e-94), CDCA8 (cor = 0.79, *P* = 8.04e-115), TPX2 (cor = 0.786, *P* = 4.1e-113), ERCC6L (cor = 0.742, *P* = 2.57e-94), BIRC5 (cor = 0.726, *P* = 1.7e-88), KIF20A (cor = 0.731, *P* = 4.4e-90), GTSE1 (cor = 0.751, *P* = 6.11e-98), CENPA (cor = 0.781, *P* = 8.46e-111), ORC6 (cor = 0.653, *P* = 4.54e-66),AURKA (cor = 0.667, *P* = 7.06e-70) (**Figures 5C–L**).

### Association between NEIL3 methylation and prognosis of KIRC patients

**Figure 6:**
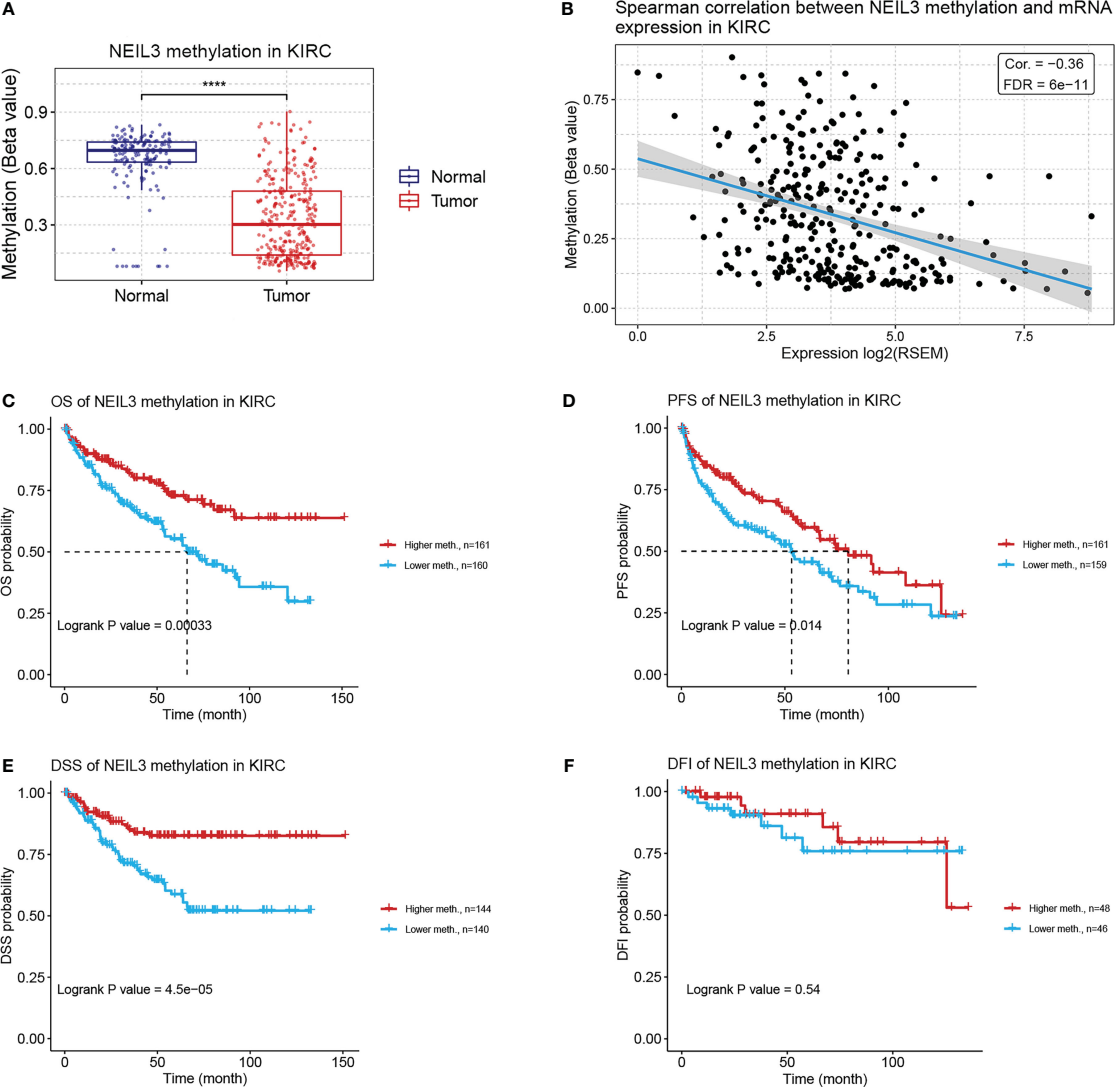
Correlation analysis between NEIL3 methylation and KIRC patients *via* the GSCA database. **(A)** The methylation level of NEIL3 between renal clear carcinoma tissues and normal renal tissues. **(B)** The correlation between NEIL3 methylation and mRNA expression. **(C–F)** The effect of NEIL3 methylation on overall survival (OS), progression-free survival (PFS), disease-specific survival (DSS), and disease-free interval (DFI) of patients with KIRC. ****, *P* < 0.0001.

Previous studies have illustrated that aberrant DNA methylation is closely related to the development and progression of multiple tumors (28, 29). In our study, we found that the methylation level of NEIL3 in renal clear carcinoma tissues was lower than that in normal renal tissues (**Figure 6A**). Spearman correlation analysis showed that there was a negative correlation between NEIL3 methylation and mRNA expression in KIRC (cor = −0.36, FDR = 6e-11) (**Figure 6B**). Furthermore, we found that compared with the hypermethylated patients, NEIL3 hypomethylated patients had worse OS, progression-free survival (PFS) and DSS, while there was not significantly difference in disease-free interval (DFI) between the two groups (**Figures 6C–F**). Overall, patients with NEIL3 hypomethylation (indicating higher expression of NEIL3 mRNA) had worse survival outcomes than patients with NEIL3 hypermethylation.

### Relationship between NEIL3 expression and immune infiltration

**Figure 7:**
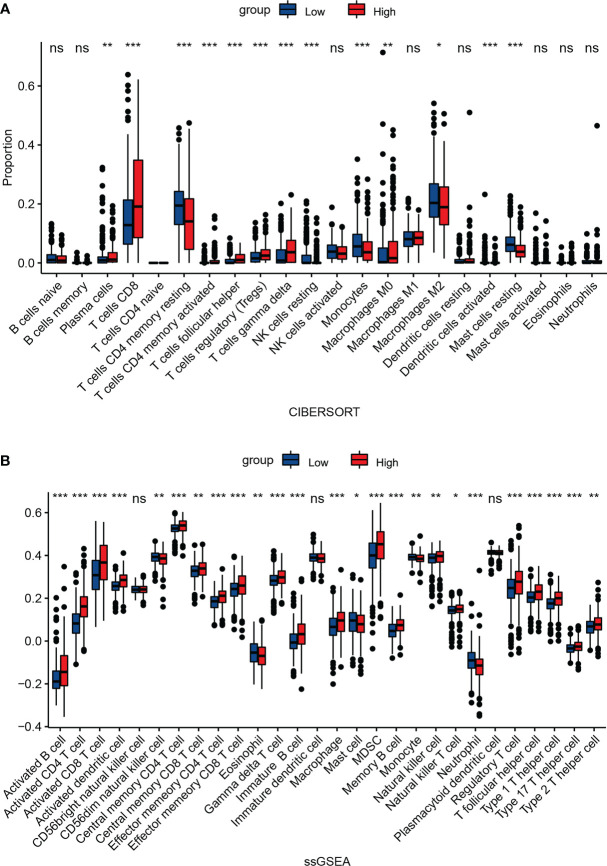
Association between NEIL3 expression and immune infiltration level. Comparison of the proportion of 22 immune cells **(A)** and expression levels of 28 immune cells **(B)** between NEIL3-high and -low groups by CIBERSORT and ssGSEA, respectively. ns, *P* ≥ 0.05; *, *P* < 0.05; **, *P* < 0.01; ***, *P* < 0.001.

In order to explore the association between NEIL3 expression and immune infiltration, we performed CIBERSORT and ssGSEA. CIBERSORT analysis showed that the proportion of plasma cells, T cells CD8, follicular helper T cells (Tfhs), regulatory T cells (Tregs), and M0 macrophages was increased in the high expression group, whereas the fraction of resting NK cells, monocytes, and M2 macrophage was decreased in the high expression group (**Figure 7A**). Meanwhile, ssGSEA revealed that activated CD4 T cells, activated dendritic cells (DCs), activated CD8 T cells, macrophages, myeloid-derived suppressor cells (MDSCs), natural killer cells (NK cells), Tfhs and Tregs were highly expressed in the NEIL3 overexpression group (**Figure 7B**). Furthermore, the connection between NEIL3 co-expressed genes and immune infiltration in KIRC was verified by ssGSEA. It was found that the expression levels of NEIL3 co-expressed genes had a positive association with multiple immune infiltrating cells (including B cells, CD4 T cells, CD8 T cells, DCs, MDSCs, Tfhs, and Tregs, etc.) (**Supplementary Figure 1**).

**Figure 8:**
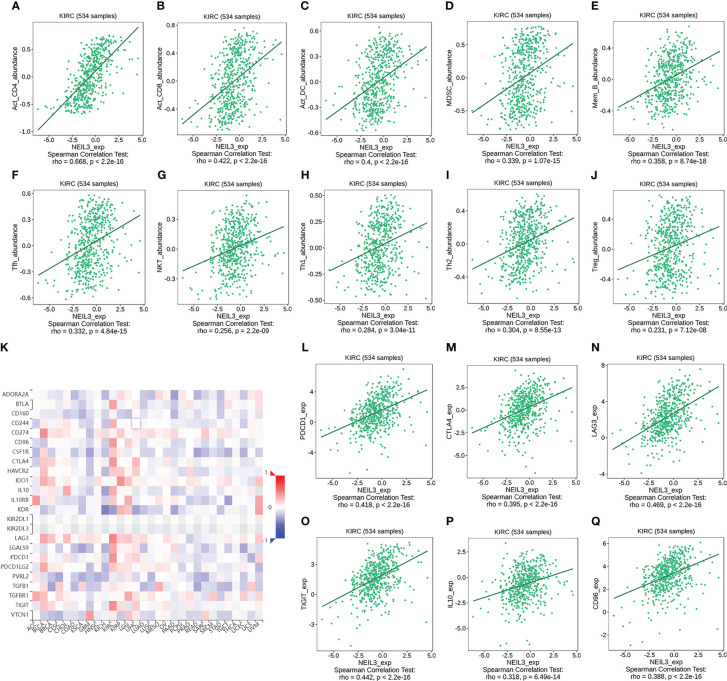
**(A–J)** Correlation analysis between the abundance of tumor-infiltrating lymphocytes (TILs) and NEIL3 expression using the TISIDB database. The results show that NEIL3 expression is positively correlated with activated CD4 T cells (r = 0.668, *P* < 2.2e-16), activated CD8 T cells (r = 0.422, *P* < 2.2e-16), activated dendritic cells (DCs) (r = 0.4, *P* < 2.2e-16), myeloid-derived suppressor cells (MDSCs) (r = 0.339, *P* = 1.07e-15), memory B cell (r = 0.358, *P* = 8.74e-18), follicular helper T cells (Tfhs) (r = 0.332, *P* = 4.84e-15), NKT cells (r = 0.256, *P* = 2.2e-09), Type 1 T helper cell (Th1 cells) (r = 0.284, *P* = 3.04e-11), Type 2 T helper cells (Th2 cells) (r = 0.304, *P* = 8.55e-13) and regulatory T cells (Tregs) (r = 0.231, *P* = 7.12e-08). **(K)** The heatmap reveals the association between NEIL3 expression and 24 immunoinhibitors across multiple tumors. **(L–Q)** Scatterplots of the correlations between NEIL3 expression and immunomodulators of PDCD1, CTLA4, LAG3, TIGIT, IL10, and CD96 in KIRC are shown. PDCD1, Aliases for PD1 Gene.

We further verified the relation between the expression of tumor-infiltrating lymphocytes (TILs) and NEIL3 *via* the TISIDB database. The results indicated that the abundance of activated CD4 T cells (r = 0.668, *P* < 2.2e-16), activated CD8 T cells (r = 0.422, *P* < 2.2e-16), DCs (r = 0.4, *P* < 2.2e-16), MDSCs (r = 0.339, *P* = 1.07e-15), memory B cell (r = 0.358, *P* = 8.74e-18), Tfhs (r = 0.332, *P* = 4.84e-15), NKT cells (r = 0.256, *P* = 2.2e-09), Type 1 T helper cell (Th1 cells) (r = 0.284, *P* = 3.04e-11), Type 2 T helper cell (Th2 cells) (r = 0.304, *P* = 8.55e-13) and Tregs (r = 0.231, *P* = 7.12e-08) was positively correlated with NEIL3 expression (**Figures 8A–J**). In addition, we divided 265 renal clear cell carcinoma samples from GSE73731 into two groups based on the median NEIL3 expression level. CIBERSORT and ssGSEA were used to assess the relationship between NEIL3 expression and the extent of immune infiltration. Similarly, we found a higher level of immune cell infiltration in the over expressed group (**Supplementary Figure 2**).

Overall, the high-expression group of NEIL3 had stronger immune infiltration than the low-expression group, especially regarding CD4 T cells, CD8 T cells, Tfhs and Tregs.

### Association between NEIL3 expression and immunoinhibitors

Finally, we investigated the association between 24 immunoinhibitors and NEIL3 expression using the TISIDB database. The heatmap showed that more than half of 24 immunoinhibitors were positively related to NEIL3 expression in KIRC (**Figure 8K**). Among them, the expression of PD1, CTLA4, LAG3, TIGHT, IL10, and CD96 was significantly associated with NEIL3 expression (all spearman’s r ≥ 0.3, all *P* < 0.001) (**Figures 8L–Q**).

## Discussion

This study revealed that the expression of NEIL3 was significantly increased in KIRC, and it was related to the histologic grade, pathologic stage, depth of invasion, distant metastasis, and vital status of KIRC patients, and that overexpression of NEIL3 was linked to worse OS, DSS, and PFI. Previous research has confirmed that NEIL3 has a crucial role in DNA repair (9), and it is related to adverse outcomes of several tumors, including hepatoma, lung cancer, and melanoma (11, 12, 30). In addition, our study showed that the increased expression of NEIL3 in KIRC tumors may be caused by its hypomethylation, and the OS, PFS, and DSS of NEIL3 hypomethylation patients are worse than those with hypermethylation patients. It has been confirmed that DNA methylation can modulate the transcription of oncogenes in human tumors (29). DNA methylation can serve as a biomarker for the prognosis of common cancers (31), supporting our finding that KIRC patients with hypomethylated NEIL3 may have worse survival outcomes.

The GO analysis has indicated that NEIL3 is involved in mitotic nuclear division, acute inflammation, kinetochore and chromosome condensation, etc., and it has been proven that tumor cells can resist the attack of immune cells through higher expression of HLA-G and PD-L1 during mitosis, suggesting that the mitotic index has an essential impact on cancer immunotherapy (32). Acute inflammatory response can stimulate the maturation and antigen presentation of DCs, thus affecting the anti-tumor immune response (33). Furthermore, GSEA analysis has shown that NEIL3 is associated with multiple DNA damage repair pathways. It is know that DNA damage response involve DNA repair, cell cycle, replication stress response, and apoptosis mechanisms (34). Base excision repair, homologous recombination, and mismatch repair are three pathways of DNA damage repair. If the DNA damage response pathway is disrupted, it will increase the risk of cancer (34). For example, the mutations of BRCA gene can lead to homologous recombination defects, which can make cells more sensitive to ionizing radiation and chemical drugs, and increase the incidence of breast, ovarian and prostate cancer (35). Additionally, a recent study reported that mutant P53 can impair the innate immune response and promote the tumor microenvironment (36).

In addition, we identified immune-related signal pathways, including antigen processing and presentation, primary immunodeficiency, and intestinal immune network for IgA production. Tumor immune escape is closely related to the absence of antigen presentation mechanism caused by loss of HLA heterozygosity or β2M mutation (37–39). HLA is primarily involved in the processing and presentation of tumor antigens, and it is essential for CD8+T cells to recognize tumors. McGranahan N has demonstrated that loss of HLA heterozygosity is a common immune escape mechanism in the evolution of lung cancer (37). A study has shown that β2M mutation can lead to the loss of expression of MHC I, and affect T cell recognition (39). It has been proved that the intestinal immune network producing IgA is essential in the occurrence of kidney carcinogenesis (40). Moreover, primary immunodeficiency may increase the risk of cancer development, especially lymphoma (41). Overall, NEIL3 is involved in diverse oncogenic and immunologically relevant biological processes and signal pathways and has the potential to be a promising target for KIRC.

In recent years, RCC has been regarded as a highly immunopermeable tumor, and studies have found that tumor immune infiltration is closely related to renal carcinogenesis (42, 43). There is increasing evidence that innate immune cells (macrophages, DCs, MDSCs, and NK cells) a nd adaptive immune cells (T and B cells) in the tumor microenvironment (TME) are closely linked to immune escape and tumor progression (44). Our results showed that the expression of NEIL3 was positively correlated with the abundance of T cells, macrophages, DCs, MDSCs, and NKT cells. Previous studies have shown that KIRC is the tumor type with the highest infiltration of T cells, and the survival rate of KIRC subsets with the highest T-cell accumulation is the worst (45). In contrast to other carcinomas, in KIRC, it is reported that the infiltration level of CD8 T cells is associated with unfavorable outcomes (46, 47). In addition, high expression of Th2 cells and Tregs was shown to be associated with worse outcomes in KIRC patients (48). A previous study showed that B-lineage cells were associated with adverse outcomes and distant metastasis in KIRC (49). Current evidence has already shown that DCs can induce tumor immune surveillance and promote immune escape (50). MDSCs derived from myeloid progenitor cells can suppress the anti-tumor activity of T cells and NK cells, and promote immune escape. It can induce epithelial-mesenchymal transition (EMT), promote tumor progression and metastasis, and promote angiogenesis (51). In addition, a meta-analysis has indicated that MDSCs are significantly correlated with poor outcomes of solid tumors, which has a good prognostic value (52).

Finally, we found that NEIL3 had positive connections with the expression of co-inhibitory molecules (such as PD1, CLTA-4, LAG-3, TIGIT, IL10, and CD96). Previous studies have shown that high expression of CTLA4, LAG3, and TIGIT is detrimental to the survival of KIRC patients (43). In addition, studies have identified the synergistic effect of LAG3 and PD1 in mediating T-cell exhaustion, thereby weakening anti-tumor activity (53). IL-10 is an immunosuppressive cytokine, which can promote T-cell exhaustion by regulating the expression of inhibitory receptors on TILs, thereby limiting effective anti-tumor immunity (54). Moreover, several studies have found that increasing IL-10 expression levels correlate with poor outcomes in cancer patients (55). These results suggest that NEIL3 has the potential to modulate tumor immune microenvironment and promote KIRC progression, but its direct mechanism requires further investigation.

Conclusively, our findings suggest that NEIL3 is highly expressed in KIRC, and it is associated with a worse prognosis. Moreover, higher NEIL3 expression is connected with more extensive immune cell infiltration. Thus, NEIL3 might be a promising biomarker for KIRC.

## Data availability statement

The original contributions presented in the study are included in the article/**Supplementary Material**. Further inquiries can be directed to the corresponding author.

## Ethics statement

Since all KIRC data were obtained from the TCGA and GEO databases, ethical approval and informed consent were not required.

## Author contributions

All authors contributed to the study’s conception and design. Conceptualization, methodology, data curation, formal analysis, visualization, and writing-original draft preparation were performed by XS. Supervision, writing-reviewing, and editing were performed by PL. All authors contributed to the article and approved the submitted version.
