# Supplementary material for: Prognostic biomarker NEIL3 and its association with immune infiltration in renal clear cell carcinoma
Source: Front Oncol. 2023 Feb 2;13:1073941. doi: 10.3389/fonc.2023.1073941 (PMC9932331; doi:10.3389/fonc.2023.1073941)
Supplement: Supplementary file 1 [file DataSheet_1.docx]

**Supplementary Figures**


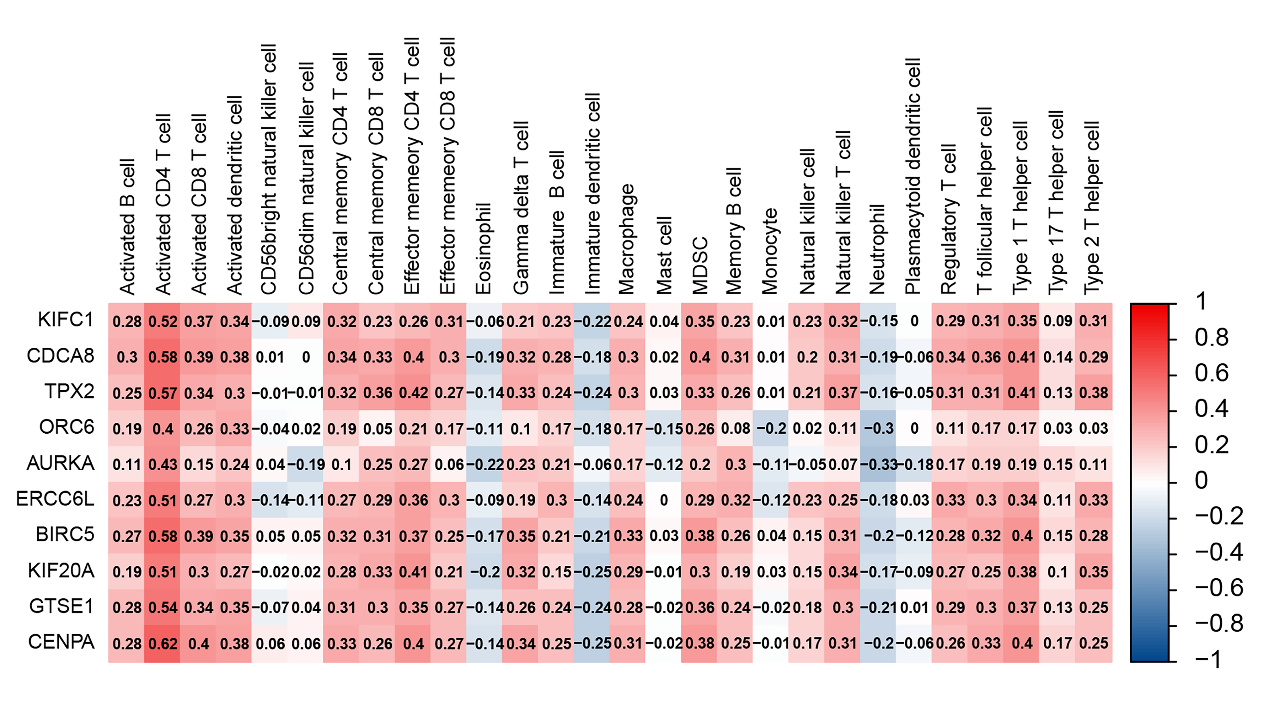


**Supplementary Figure 1.** **Correlation between NEIL3 co-expressed genes and immune infiltrating cells.** The expression levels of NEIL3 co-expressed genes were found to be positively correlated with the abundance of multiple immune infiltrating cells (such as activated B cells, activated CD4 T cells, activated CD8 T cells, activated DCs, MDSC, Tfhs, and Tregs) by ssGSEA analysis.


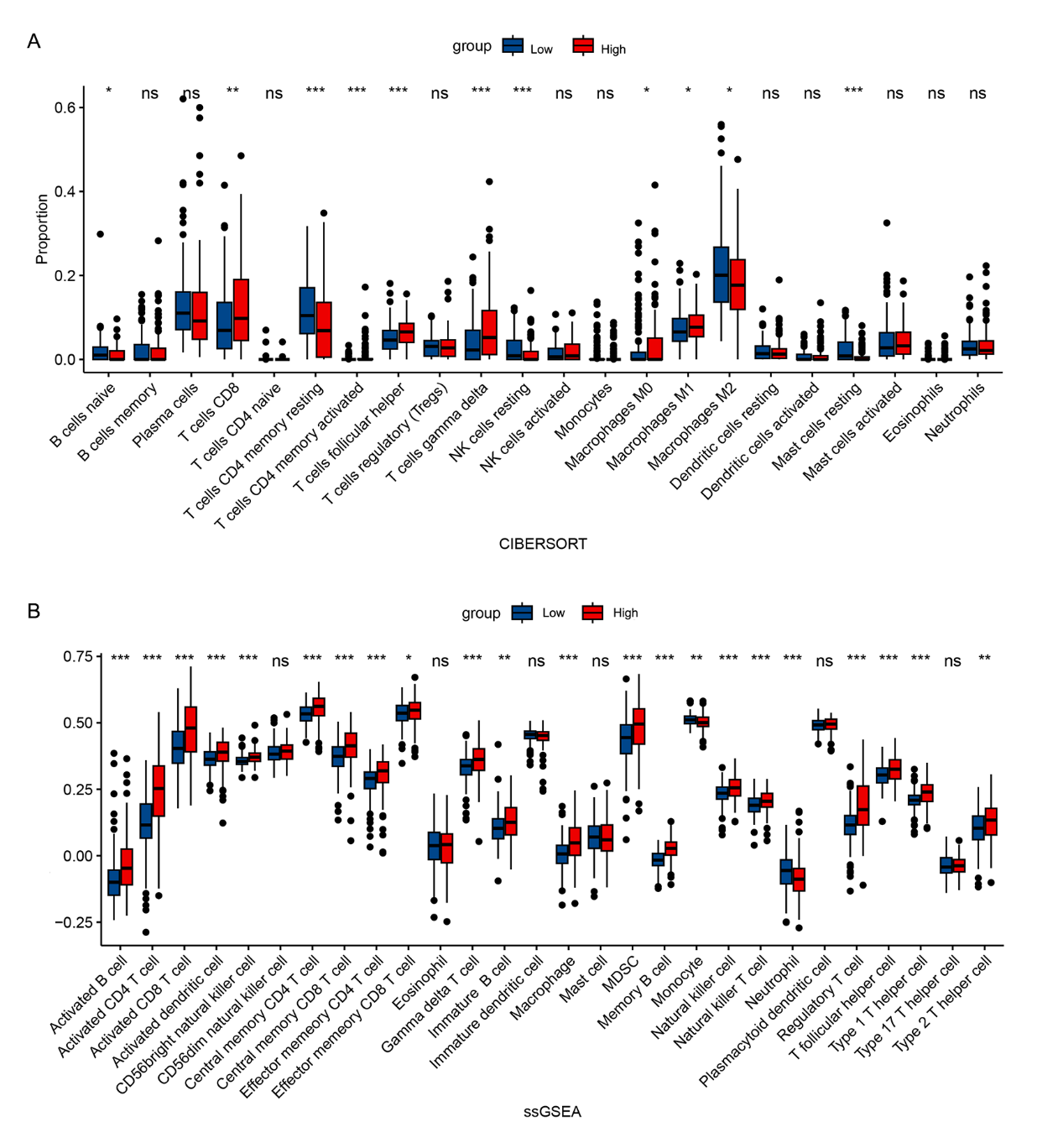


**Supplementary Figure 2.** GSE73731 validation of the relationship between NEIL3 expression and immune infiltration. Comparison of proportion of immune cells (A) and expression of immune cells (B) between NEIL3 high and low expression groups. ns, *P* ≥ 0.05; *, *P* < 0.05; **, *P* < 0.01; ***, *P* < 0.001.
